# Sirolimus-Versus Zotarolimus-Eluting Stents in Acute Coronary Syndromes With C Type Left Anterior Descending Artery Lesions: A Three-Year Clinical Follow-Up

**DOI:** 10.4021/cr170w

**Published:** 2012-05-20

**Authors:** Seher Gokay, Davran Cicek, Haldun Muderrisoglu

**Affiliations:** a Department of Cardiology, Baskent University School of Medicine, Saray Mah, Yunusemre cad, No: 1, 07400, Antalya, Turkey; b Department of Cardiology, Baskent University School of Medicine, Bahcelievler, No: 1, Ankara, Turkey

**Keywords:** Major adverse cardiac event, Stent thrombosis, C-type lesion, Drug-eluting stent

## Abstract

**Background:**

Drug-eluting stents have improved the efficacy of percutaneous coronary intervention and made it the preferred therapy in the treatment of ischemic heart diseases including acute coronary syndromes. The objective of the study was to compare the clinical efficacy and safety of sirolimus-eluting stent with that of zotarolimus-eluting stent following percutaneous coronary intervention for acute coronary syndrome patients with C-type left anterior descending stenosis.

**Methods:**

A total of 154 acute coronary syndrome patients with C-type lesions in the left anterior descending artery, requiring a stent > 28 mm in length, were randomized into two groups to receive either sirolimus- (n = 74) or zotarolimus-eluting stent (n = 80). The follow-up period after stent implantation was approximately 36 months. The primary endpoint was a major cardiac event (a composite of cardiac death, myocardial infarction, or ischemia-related target vessel revascularization), and the secondary endpoint included these individual end points plus stent thrombosis.

**Results:**

After 3 years follow-up, the rate of the primary end point (major cardiac event: cardiac death, myocardial infarction, ischemia-related target vessel revascularization) was 16% in the sirolimus group (n = 12) versus 11.2% in the zotarolimus group (n = 9) (P = 0.2). Although there were four cases of stent thrombosis with sirolimus-eluting stent and one with zotarolimus-eluting stent (4.0% sirolimus vs. 1.25% zotarolimus; P = 0.2), neither non-Q myocardial infarction (4.0%sirolimus vs. 1.25% zotarolimus; P = 0.2) nor stent thrombosis, differed significantly.

**Conclusions:**

Although zotarolimus-eluting stent implantation showed more favorable results with respect to stent thrombosis and major adverse cardiac event rates compared to sirolimus-eluting stent implantation, statistically, both stent groups have nearly similar clinical safety and efficacy in the treatment of acute coronary syndromes with C-type lesions in the left anterior descending artery disease.

## Introduction

Coronary revascularization (percutaneous and/or surgery) is the major treatment of patients presenting with acute coronary syndromes (ACS) which is a common manifestation of atherosclerotic dis­ease. The advent of the bare metal stents (BMS) and the following drug-eluting stents (DES) have improved the efficacy of percutaneous coronary intervention (PCI), and made it the preferred therapy in the treatment of ischemic heart diseases including ACS [1]. Nevertheless, the treatment of obstructive lesions, especially those in the left anterior descending coronary artery (LAD), represents a challenge for interventional cardiologists due to the wide heart area LAD supplies [2]. It has been shown that percutaneous revascularization of the proximal LAD with implantation of DES is a safe and very efficient therapeutic strategy in the short and long terms [2]. Sawhney et al state that LAD intervention with sirolimus-eluting stents (SES) significantly reduces angiographic restenosis and clinical events compared with BMS [3]. However, stent thrombosis (ST) following PCI, still remains a fearing outcome of ACS undergoing PCI, and has raised some questions about the long-term risks of the DES [4]. This concern has led to the emerging of numerous studies aiming to provide some information about the differences between the various DESs with regard to stent thrombosis. Since the first generation DES turned out to have the potential for late ST, second-generation DES were developed with hopes for improved efficacy and/or safety.

Although there is a plethora of DES trials regarding native coronary artery lesions, there are limited data comparing the first and second generation DES for the treatment of ACS patients with C type LAD lesions. The present study was conducted in the light of the conflicting results of some trials where sirolimus-eluting (SES; Cypher) and zotarolimus-eluting (ZES; Endeavor) stents were used in the treatment of coronary artery disease. The objective was to compare the clinic and angiographic outcomes of SES as a first generation DES, and ZES as a second generation DES. The authors hope to have made some contribution to the decision making process with regard to the choice of drug eluting stents for the treatment of ACS patients with C type native LAD lesions.

## Methods

### Study design and patient population

This prospective study was conducted from February 2005 to March 2008. The duration of follow-up was 4 years and here we report the 3 years outcome. The study population was comprised by 154 ACS patients with C-type LAD disease undergoing implantation of an average of 31 ± 3 mm Sirolimus- (n = 74) (SES; CYPHER; Cordis Corporation, Johson and Johnson, Miami Lakes, Florida) or 33 ± 5 mm long Zotarolimus- (n = 80) (ZES; Medtronic Vascular, Santa Rosa, CA) eluting stent. Patients were eligible if they had a history of unstable angina (UA) and signs of myocardial ischemia. Patients were also required to have a de-novo target lesion in the LAD of 51% to 99% stenosis needing a stent > 28 mm in length (visual angiographic estimates). Exclusion criteria were patients with CAD history (prior PCI, prior MI, and prior CABG), chronic total occlusion, and lesions needing a < 28 mm stent.

### Study procedure and angiographic analysis

Written informed consent was obtained from all subjects, and the study protocol was approved by the local ethical committee. The angiographic inclusion and exclusion criteria were reassessed after initial angiography at the start of the procedure. All C-type LAD lesions were identified and recorded as such. Cineangiograms were analized using a validated edge system (CMS, version 5.2, MEDIS, Leiden, and the Netherlands). Then patients were randomly assigned in a single-blinded manner for treatment with a SES or a ZES.

All patients received aspirin (at least 100 mg once daily) and clopidogrel 75 mg once daily, or ticlopidine 250 mg twice daily at least three days before the procedure, with a loading dose of 300 mg of clopidogrel to patients not pretreated. Unfractionated heparin (UH) was administered at the beginning of the procedure at the dose of 100 IU/kg to achieve an activated clotting time > 250 s. The patients received intracoronary nitroglycerin (0.1 to 0.2 mg) before initial and final coronary angiograms to achieve maximal vasodilatation. Glycoprotein (GP) IIb/IIIa inhibitors were administered at the discretion of the operator. All patients were maintained at antiplatelet therapy after the procedure (aspirine 300 mg /d for 3 months, then 100 mg/d infinitely; clopidogrel 75 mg/d for 6 to 12 months). The PCI procedure and stent implantation were performed using standard methods, through a femoral or radial approach. A C-type lesion was defined as a diffuse (> 2 cm length), excessive tortuosity of the proximal segment, extremely angulated (> 90 degrees), inability to protect major side branch [5]. Lesions were treated in accordance with standard PCI guidelines. Pre- and post-dilatation, and the stent of choice (i.e., SES or ZES) were left at the discretion of the operator. The control coronary angiographies were performed when there was evidence of ischemia. All patients had either UA or a myocardial infarction (MI) (ST-segment elevation or non-ST-segment elevation). ACS consisted of unstable angina pectoris, I-III B according to Braunwald’s classification [6], and AMI. AMI was defined as typical chest pain, electrocardiographic changes and creatine kinase elevation to twice the upper limit of normal.

Angiographic success was defined as Thrombolysis in Myocardial Infarction (TIMI) flow grade III and < 30% residual diameter stenosis by visual assessment. Restenosis was defined as > 50% stenosis in diameter by qualitative coronary angiography within a previously stented segment. For the assigned study stent, device success was defined as ≤ 50% diameter stenosis of the target lesion, and procedural success was defined as device success with no in-hospital major adverse coronary event (MACE). Angiography was scheduled at six months or earlier if clinically indicated.

### Clinical follow-up, definitions and study end points

Clinical follow-up was performed by either telephone contact or office visits at 1, 6, 12, 24, 36 months. All patients were invited for repeat coronary angiography between 6, 12 and 36 months; however, participation in angiographic follow-up was not mandatory for inclusion in the study. Relevant data were collected and entered into a computerized database by specialized personnel at the cardiovascular interventional heart center. Patients were asked specific questions about the development of angina according to Braunwald Classification of unstable angina. They were also monitored for (MACE), a composite end point comprising cardiac death, myocardial infarction (MI), and target vessel revascularization (PCI/CABG).

Intraprocedural stent thrombosis was defined as an angiographically confirmed intraluminal filling defect within the stent resulting in TIMI anterograde flow grade 0 or I that occurred during the procedure. Postprocedural ST was defined as any of the following between the end of the procedure and the end of follow-up: angiographic documentation of stent occlusion, unexplained sudden death when the stent was not known to be patent, or MI or urgent target lesion revascularization occurring in the territory of LAD. Target vessel revascularization was defined as either percutaneous or surgical revascularization (CABG) of stented epicardial vessel.

The secondary end point was stent thrombosis. Stent throbosis was classified based on the time elapsed since implantation. Stent thrombosis occurring during the stenting procedure or within the subsequent 24 hrs was defined as acute ST, subacute ST - between 1 and 30 days after implantation, late ST-between 1 month and 1 year, and very late ST-more than 1 year after the procedure. The definitions of MI and ST used in the study were consistent with the newest consensus of the Academic Research Consortium [7]. All primary and secondary clinical end points were adjudicated by an independent clinical events committee blinded to the patient’s treatment assignment.

### Statistical analysis

All statistical analyses were performed with SPSS for Windows (version 10.0, Chicago, USA, Categorical variables are presented as percentages or proportions, and continuous variables as mean values ± SD. Comparison of continuous variables was performed with unpaired t-tests (normal distribution) and the nonparametric Mann-Whitney test (skew distribution). Analysis of categorical variables was done with Fisher’s exact test and chi^2^ test. We used the Kaplan-Meier time-to-event estimates for the primary events at 24-month follow-up. With the Kaplan-Meier method and log-rank test, we compared the difference between the SES and the ZES cohorts. A P value < 0, 05 was considered statistically significant.

## Results

Between February 2005 and March 2008, a total of 154 patients were enrolled in the study and randomly assigned to receive ZES (80 patients) or SES (74 patients). Baseline clinical characteristics of patients were similar among the groups (Table 1). In the SES group there were 79.7% of UAP, and 20.2% of MI. Similarly, in the ZES group there were 75% of UAP and 25% were AMI.

**Table 1:** Baseline Patient Clinical Characteristics

	Sirolimus-group (n = 74)	Zotarolimus-group (n = 80)	P value
Age* (years)	59 ± 9.2	61 ± 10.2	0.4
Diabetes mellitus n (%)	32 (43.2%)	39 (48%)	0.3
Hypertension n (%)	43 (58%)	51 (63.7%)	0.6
History of smoking n (%)	48 (64%)	40 (50%)	0.3
Hyperlipidemia n (%)	44 (59%)	59 (73.7%)	0.3
LVEF (66.9 ± 5.5%)	67.5 ± 4.8	66.4 ± 6.2	0.4
USAP n (%)	59 (79.7%)	60 (75%)	0.4
MI n (%)	15 (20.2%)	20 (25%)	0.4
T.Cholesterol (mg/dL)*	211.6 ± 48.4	231.4 ± 54.3	0.8
LDL (mg/dL)*	148.3 ± 46.7	149.5 ± 47.7	0.5
HDL (mg/dL)*	36.3 ± 7.6	36.1 ± 7.9	0.5
TG (mg/dL)*	164 ± 101.7	164.2 ± 103.5	0.7
Glucose (mg/dL)*	144.1 ± 61.8	138.5 ± 45.6	0.2

*Data expressed mean ± SD, P < 0.05 accepted statistically significant. LVEF: Left ventricular ejection fraction; MI: Myocardial Infarction; PTCA: percutaneous revascularization; USAP: Unstable angina pectoris.

Angiographic characteristics of patients in the two groups were also similar (Table 2). The rates of device success and treatment success were similar: 100% for both study groups. Average stent length was 31 ± 3 mm for the SES, and 33 ± 5 mm for the ZES group (P = 0.2).

**Table 2:** Angiographic Characteristics of Patiens in Two Groups

	Sirolimus-group (n = 74)	Zotarolimus-group (n = 80)	P value
Stent (n)	84	92	0.2
Stent diameter (mm)	29 ± 5	28 ± 7	0.8
Stent length (mm)	31 ± 3	33 ± 5	0.2
Lesion length (mm)	27 ± 2	26 ± 4	0.1
Max. implantation pressure (atm)	16	18	0.2
Angiographic success n (%)	74 (100%)	80 (100%)	0.5

Data expressed mean ± SD, P < 0.05 accepted statistically significant.

Comparison of acute, subacute, late and very late stent thrombosis in groups were shown in Table 3. Among 154 patients 5 developed stent thrombosis, and female/male ratio was 4/1. The incidence of acute, subacute, late and very late stent thrombosis at 36 months was higher in the SES cohort (statistically insignificant). In the SES group one patient suffered from MI due to acute stent thrombosis, while no acute stent thrombosis was observed in the ZES group (1.35% vs 0%). Similarly, one patient experienced subacute, one late, and one very late stent thrombosis in the SES group. There was no case of late and very late stent thrombosis in the ZES group. Only one patient experienced unstable angi UAP due to subacute ST in the ZES group. Subacute and late STs seen in the SES group were clinically UAP, acute and very late ones were clinically AMI. Patients who experienced subacute, late and very late stent thrombosis in the SES group were cigarette smokers. All patients in the SES group had HT. DM was observed in most stent thrombosis cases except for the single subacute ST case in the SES group. In the SES group the patient who experienced subacute ST was 49 years old. In contrast, the other ST thrombosis patients were over 60. All patients were taking clopidogrel and aspirin. The patient experiencing subacute ST in the ZES group had received GpIIb/IIIa infusion but the others STs did not. Overall, only one patient experienced subacute ST in the ZES group with a stent 3.5 mm in diameter and 38 mm in length.

**Table 3:** Comparison of Acute, Subacute, Late and Very Late Stent Thrombosis in Groups

	Acute	Subacute		Late	Very late
Patient n (%)	1 (1.25%)	2 (1.29%)		1 (1.35%)	1 (1.35%)
Age	69	68	49	68	66
M/F	F	F	F	M	F
Clinic	Acute MI	UAP	UAP	UAP	Acute MI
Smoking	-	-	+	+	+
Hypertension	+	-	+	+	+
DM	+	+	-	+	+
Stent type	Cypher	Endeavour	Cypher	Cypher	Cypher
Stent length	28 mm	38 mm	33 mm	33 mm	33 mm
Stent diameter	2.75 mm	3.5 mm	2.75 mm	3.0 mm	2.5 mm
Clopidogrel*	+	+	+	+	+
Aspirin	+	+	+	+	+
GpIIb/IIIa	-	+	-	-	-

MI: Myocardial Infarction; UAP: Unstable angina pectoris.

The clinical characteristics of the patients at three years follow-up are reported in Table 4. At 36 months, the incidence of MACE (cardiac death, myocardial infarction (MI), and target vessel revascularization) was 11.2% (n = 9) in the ZES cohort and 16% (n = 12) in the SES cohort (P = 0.258). The rate of non-Q-wave MI was higher in the SES group than in the ZES group, but the difference was statistically insignificant (4.0% vs. 1.25%; P = 0.2, respectively). Also, there were no major differences in the rates of coronary artery bypass graft (CABG) procedures (2.7% vs. 2.5%; P = 0.5), TVR (4.0% vs. 2.5%; P = 0.3), non-TVR (2.7% vs. 2.5%; P = 0.1), Q-wave MI (2.7% vs. 2.5%; P = 0.5), cardiac death (2.7% vs. 2.5%; P = 0.5).

**Table 4:** Clinical Characteristics of Follow-Up Patients at Three Years

	Sirolimus-group (n = 74)	Zotarolimus-group (n = 80)	P value
Revascularization PCI n (%)			
target vessel	3 (4%)	2 (2.5%)	0.3
non target-vessel	2 (2.7%)	2 (2.5%)	0.1
CABG n (%)	2 (2.7%)	2 (2.5%)	0.5
Myocardial infarction n(%)			
Q-wave	2 (2.7%)	2 (2.5%)	0.5
non-Q-wave	3 (4%)	1 (1.25%)	0.2
Cardiac Death n (%)	2 (2.7%)	2 (2.5%)	0.5
MACE n (%)	14 (18%)	11 (13%)	0.2

CABG: Coronary artery bypass grafting; MACE: Major adverse cardiac event; PCI: percutaneous coronary intervention.

## Discussion

Percutaneous treatment has shown to improve clinical outcome in the symptomatic coronary artery disease by resolving coronary obstruction [1]. Moreover, percutaneous intervention of LAD lesions with sirolimus-eluting stent has been reported to result in revascularization rates comparable with historic single-vessel CABG revascularization rates [3]. Accordingly, PCI with drug-eluting stents is the current treatment of choice for patients with isolated proximal left anterior descending coronary artery disease [8].

However, in the DES area, especially where restenosis is of less issue, ST is considered the Achilles’ heel of PCI [9]. Although the incidence and timing of ST occurring between 30 days and 1 year (late ST (LST)) were reported to be similar after both bare metal stent (BMS) and DES implantation, ST beyond 1 year after stent implantation (very LST (VLST)) was reported to occur more frequently after DES implantation than after BMS implantation [10]. Some data suggest that increased LST risk may be due to delayed arterial healing with incomplete re-endothelialization and/or a chronic inflammatory response [11-13]. Although this excess risk appears to be small and does not translate into adverse clinical outcome, this has remained a concern, especially in ACS patients [4].

Although it has been shown that first-generation DESs are safe and efficacious for both on-label and off-label use when implanted in the native circulation [14], concerns related to increased propensity for late and very late ST has led to the development of second-generation drug-eluting stents. Second-generation stents have different drugs, lower drug doses, and newer stent designs - particularly, thinner struts and newer, more biocompatible or even bioabsorbable polymers [15]. The development of second-generation DES along with prolonged dual antiplatelet therapy led to ST risk reduction. They differ mainly in polymer technology and metallic stent structure [4].

There is compelling clinical evidence that ZES carries an extremely low risk of late ST [16]. A prospective, randomized trial showed that vasoconstriction in response to Ach in the peri-stent region was less pronounced in the ZES group than the SES group at 6-month follow-up, which suggests that endothelial function associated with ZES can be more preserved than with SES [17]. Accordingly, the Endeavor studies [18-20] provided evidence that the ZES was safe and efficacious, and because of exceedingly low reported rate of ST with the ZES platform it was suggested that there may be an advantage of using ZES in acute, high risk PCI such as STEMI. Our results were consistent with those of the Endevour studies in the way we found low ST rates in ZES group: that is, there was only one patient clinically experiencing UAP in the setting of subacute ST in contrast to a total of four STs in the SES group (one acute, one subacute, one late and one very late).

Until recently, the second generation DESs had proved to be significantly more effective and safe compared to the first-generation DESs in reducing the risk of ST in ACS patients owing to a better design, greater biocompatibility with release kinetic [4]. However, the SORT OUT III trial showed an increased risk of ST as well as an increased risk of MACE and TLR in ZES compared to SES, with no difference in all cause mortality. Implantation of ZESs compared to SESs is associated with a considerably increased risk of adverse events in patients with diabetes at 18-month follow-up [21].

The results of the SORT OUT III differ from those observed in Endeavor clinical trial program in the sense that the SORTOUT III study included patients with complex lesions, such as bifurcations, ostial lesions, left main lesions, long lesions and chronic total occlusions, as well as patients with ACS and STEMI, and that it was powered to address clinical points. In the present study, MACE rate was significantly higher in the SES patients. The patients in our trial were in some features similar to those of in the SORTOUT study in that it included lesions > 2 cm, and patients with ACS and MI.

We found increased rates of non-Q myocardial infarction and stent thrombosis, in the Zotarolimus-Eluting Endeavor stent as compared to the Sirolimus-Eluting Cypher stent although the rates were statistically insignificant.

In the present study, we compared the first-generation sirolimus-eluting stent with the second-generation zotarolimus-eluting stent in ACS patients with C type LAD lesions, and found that the clinical and angiographic efficacy at 36 months was more in favor of the ZES group compared to that of the SES group. That result was consistent with Endeavor trials.

### Study limitations

The number of enrolled patients was relatively small.

### Conclusions

Despite the continuous double antiplatelet therapy one patient had ST in the ZES group and four in the SES group. Based on these results one might conclude that the ZES treatment of acute ischemic patients with C type LAD lesion is more effective than the SES treatment. Although dual antiplatelet therapy plays a key role in the prevention of ST, the type of DES might have an important impact on it. However, larger patient population studies are needed to clarify the statement in the above mentioned patient group.
